# Militarizing politics of recognition through the Invictus Games: post-heroic exalting of the armed forces

**DOI:** 10.1057/s41290-022-00172-3

**Published:** 2022-11-01

**Authors:** Brad West

**Affiliations:** Justice & Society, University of South Australia, GPO Box 2471, Adelaide, South Australia 5001 Australia

**Keywords:** Parasport, Militarization, Identity politics, Cultural recognition, Post-heroic, Contemporary veterans

## Abstract

The Invictus Games is an international sporting competition involving military veterans who have become either wounded, injured or sick during their service. Having become a prominent event in the public sphere of participating nations that are drawn from Western security alliances, this article outlines results from a thematic analysis of Australian media surrounding the 2018 Sydney Games. While reporting of the Games included the use of cultural frames that reflect traditional symbolic relationships between sport and war, the data reveal new military–civilian discourses drawn from identity politics and focused on cultural recognition. These discourses emerge through the Invictus Games by (1) disability providing a cultural basis to demand greater respect for contemporary veterans and military service; and (2) empowerment narratives of rehabilitation being symbolically connected to participants’ reengagement with their former military identity. Institutional problems central to rising political activism amongst contemporary veterans did not feature in the media coverage. It is argued that the Invictus Games illustrates the need for sociology to conceive of militarization in more multidimensional ways, appreciating both the prominence of a civilian–military gap in contemporary culture and how various social actors in Defense utilize post-heroic narratives in seeking to redress this cultural divide.

## Introduction

This article analyzes the discourses and narratives that surround the Invictus Games. Founded by Prince Harry, Duke of Sussex, who was a Captain in the British Army and served two tours of Afghanistan (2008 and 2012), the Invictus Games is an invitation based international sporting event for wounded, injured, and sick servicemen and women. Participants include those that have discharged as well as those still serving in the armed forces. The inaugural Games were held in 2014 (London) and subsequently ran in 2016 (Florida), 2017 (Toronto), 2018 (Sydney) and, due to the Covid-19 pandemic, were then delayed until the 2022 iteration (Hague). Participating countries broadly reflect Western securities alliances. Initially these were largely drawn from the post-2001 military campaigns in Afghanistan and Iraq, with the former Soviet states Ukraine and Romania added for the 2018 Games, Belgium and South Korea invited to the 2022 iteration, and Israel set to attend the 2023 Invictus Games in Dusseldorf.

Despite its short history, the Invictus Games has attained a prominent place in the public imagination of many participating nations and has attracted significant discourse in the public sphere. For example, approximately 100,000 spectators attended the 2018 Games (Invictus Foundation 2018) with the Opening Ceremony being the most watched non-news television program in Australia on the night, with the eight-day television coverage of the event reaching twenty-three percent of the metro population (Mediaweek 2018). In the United Kingdom it is estimated that prior to the recent 2022 Hague Games, over 65 million viewers had watched the competition on BBC television (Drysdale 2019). With such popularity, the Invictus Foundation that co-ordinates the Games and other related sporting events and charity activities, counts amongst its major sponsors global brands such as Land Rover, Super Dry and Major League Baseball. The narrative pull of the Invictus Games is reflected in the popular subscription streaming service Netflix commissioning Prince Harry and his celebrity actress wife Meghan Markle to produce *Heart of Invictus,* a ‘behind the scenes’ documentary of the Games (Waterson 2021). Such is the cultural significance of Invictus that following the death of Queen Elizabeth II, there were frequent references to her comedic social media appearance with Prince Harry, addressing Barack and Michelle Obama, as part of the promotion for the 2016 Invictus Games (e.g., Adam 2022).

The aim of this article is to better understand the contemporary symbolic relationship between sport and war, something commonly referred to in sociology as the sport/war nexus (Donaldson 2020; King 2008). This is achieved through a case study of the 2018 Invictus Games in Sydney. Evidence is drawn from a thematic coding of the various media content of the 2018 Games produced by the official local broadcaster, the Australian Broadcasting Corporation (ABC). Based on this data, it is argued that the Invictus Games cannot be understood merely in relation to current dominant sociological comprehensions of the sport/war nexus. While the Sydney Games were in part narrated through traditional discourses around the heroic interconnections between sport and war, the event drew heavily on the cultural rhetoric of identity politics, including as it relates to disability and parasport. In this article the term identity politics is understood in reference to societal shifts towards cultural identification not primarily with the national collective but the separate groups that constitute it. Specifically, identity politics is used in this article in reference to the belief that the dignity and welfare of groups is strongly connected to the cultural recognition provided by others (Honneth 1995; Taylor 1992). This is commonly referred to as the politics of cultural recognition. While there is a significant sociological literature on identity politics and cultural recognition in the sporting sphere, especially within parasport and the Paralympics, contemporary disability politics have not been extensively examined in relation to the sport/war nexus (cf. Caddick et al. 2021).

In the article it is argued that within the Invictus Games, identity politics discourses were subject to a process of cultural militarization by (1) disability providing a cultural basis to demand greater respect for contemporary veterans and military service; and (2) empowerment narratives of rehabilitation being symbolically connected to participants’ reengagement with their former military identity. It is contended that the discourses and narratives of the Invictus Games should be understood as reflecting the ways in which various social actors in Defense are both influenced by shifts in the civil sphere, something that has been brought about by the current civilian–military gap (Rahbek‐Clemmensen et al. 2012), and can innovatively utilize them, including to redress these societal transformations. Drawing on the strong program of cultural sociology (Alexander and Smith 2018), the article seeks to comprehend the complex and culturally contingent ways that the civil and military spheres interact in relation to the Invictus Games. In doing so the article points to analytic limitations in how the sociology of sport has conceptualized the process of militarization.

## Sport/war nexus and measuring militarization

As evident in the continued popularity of George Orwell’s dictum that sport ‘is war minus the shooting’ (1945, p. 10), there is no shortage of debate about the relationship between sport and war. While sport in its various guises has long been used as an informal form of military training, concern about the relationship between sport and militarism is distinctively modern, and closely aligned with identity fears associated with automation and urbanization. This can be seen in the emergence of ‘muscular Christianity’ that shaped the sporting heroic model of the late Victorian era (Dawson 1994; Mangan 1981), a development that fed into cultural militarism prior to WWI, particularly through promoting beliefs about ‘a coming man.’ The Nazi’s use of the 1936 Berlin Olympic Games for propaganda purposes is the more commonly cited example of the ways in which athleticism keys into fears about racial identity and national decline (Hargreaves 1992; Roche 2000). It is the ideological hostilities of the Cold War, and how these were played out through the Olympic Games and other major international sporting events (Grix and Houlihan 2014; Tomlinson and Young 2006), however, that has most significantly shaped sociological theories on the sport/war nexus (King 2016; Messner 1994; Trujillo 1995). This is not surprising as it was the 1960s and 1970s, when Western popular culture was preoccupied with Cold War themes that the sociology of sport as a sub-discipline arises. But what cultural shifts in the sport/war nexus might have emerged in the current post-Cold War era? This period has been characterized by New Wars (Kaldor 2012) that typically involve non-state actors and low intensity conflicts, rather than clear ideological divides and the experience or fear of conventional and nuclear warfare. Military strategists as well as cultural theorists have referred to the present age as being post-heroic. I use the term here to reference the intersection of two phenomena: (1) the way warfighting has shifted to reflect the reduced appetite of publics in Western nations to suffer casualties in military operations (Luttwak 1995; Scheipers 2014; Swed and Crosbie 2019); and (2) the way traditional military heroic narratives and honors have been increasingly subject to a wanning of affect (West and Crosbie 2021; King 2010; Schwartz 2008). This is not to suggest that heroism as it relates to militarism has disappeared. Rather the term is used to indicate that the traditional heroic formula is culturally marginalized or appears less often. This includes heroic figures now more likely to be assigned qualities that are relatively mundane, a popular focus on their civilian rather than institutional military identities, and with less cultural distinctions from ordinary citizens.

The sociology of sport has paid insufficient attention to the shifting civilian–military relations that define the post-heroic era. Instead, scholarship on the sport/war nexus has tended to focus on the enduring characteristics of modern militarism in sports. This has included concern with the ways in which the institutional structures and discourses of sport are dominated by the universal needs of military organization and mobilization, the enduring resonance of modern war metaphors and the cyclical reinforcement of militaristic national heroism through war commemoration (Der Derian 2009; Jenkins 2013). To the extent that changing social attitudes to war or institutional cultural shifts in the military are acknowledged, it is argued that sporting representations have not followed suit. In this formulation sport is commonly presented as hegemonic phenomena that celebrates traditional cultural forms in the face of broader societal transformations. For example, the sociology of sport has analyzed how the sport/war nexus plays out in the new post- 9/11 security environment (Fischer 2014; Schimmel 2017; Vincent et al. 2010) and in the context of new popular sporting traditions, including the rise of professional women’s sport (Batts and Andrews 2011; Bowes and Bairner 2018; Newman 2007). However, the focus of this scholarship is in pointing to the endurance and cultural adaptability of traditional militaristic cultural frames.

In this literature sport in seen as inherently supporting the interests of the military, either directly as a mirror of the military industrial complex or indirectly through naturalizing patriotism, neoliberalism, conflict, and competition (Butterworth 2017; Knoester and Davis 2021; McDonald 2020; Stahl 2010). While the military is assigned significant power in the shaping of the sport/war nexus, seldom is the military or the Defense sector institutionally analyzed in how it is shaped by shifts in civilian–military relations. This analytic limitation has been recently explored in critiques of the militarization of culture thesis (McKay 2013; Woodward et al. 2017), the perspective from which the analysis of the sport/war nexus in the sociology of sport is generally drawn. These critiques argue that the military and militarism is typically reified in sociological and political science analysis, assigning them with inherent and universal characteristics, while assuming cultural engagements with military themes automatically result in militarization.

The focus on the perceived power of military interests and ideology to shape culture also means that we find a lack of scholarship on the sport/war nexus that privileges sport as a particular and relatively independent cultural field (Bourdieu 1996) with a power to shape civilian–military relations. This is surprising given that the sociology of sport has otherwise in recent decades increasingly acknowledged the potential of sport to be a site of new political movements that actively drives social change (Woroniecka-Krzyzanowska 2020). This perspective has been particularly prominent in the analysis of alternative sporting contests and competitions, including as part of resistance to white racism and colonialist power (Biyanwila 2018; Carrington 1998), enhancing the visibility and participation of LGBTQI+ in sport (Segrave 2016) and in challenging ablest worldviews (Haslett et al. 2020).

In disability politics the growth and prominence of the Paralympics has been prominent in connecting identity politics with sport (Wedgwood 2014). Primarily the Paralympics has been influential by encouraging a comprehension of disadvantage away from social-economic status to the lived embodiment of other identities, with individual and group dignity as well as social marginalization seen as being the outcome and responsibility of the cultural recognition provided by others in society. The sociological literature on Paralympics and parasport actively engages in such identity politics debates, for example over the way the Paralympics challenges or reinforces disability stereotypes, reinforces a hierarchy of disability, nationalism, white privilege, and technological rationalism (Howe 2011; McGillivray et al. 2021; Misener 2012). Such literature also warns of the ways in which parasport can be counterproductive for disability rights and result in disabled athletes feeling disempowered (Berger 2016). However, there is little exploration of the ways in which the discourses of identity politics in parasport may influence or be utilized by the military (cf. Caddick et al. 2021).

In analyzing the media reporting of the 2018 Invictus Games, this article seeks to deploy an analytic perspective that is more open to comprehending new discourses and narratives in the sport/war nexus. These include those that might derive from new levels and types of cosmopolitan sentiment (Beck and Levy 2013; Igarashi and Saito 2014) and greater cultural sensitivity towards violence in society (Pinker 2011; Scheipers 2014). Discourses and narratives may also connect with new demographic shifts in national militaries (Hoglin 2021; Milton and Mines 2021; Vasquez and Napier 2022) and rising political activism and critiques of the Department of Defense and Veteran’s Affairs by post-2001 veterans and their families (Flores 2017; Gutmann and Lutz 2010). These representations are situated within prominent Western veteran discourses about the disenchanting aspects of military life. This includes the belief that military service contributes to marital disharmony and family tension (Kleykamp 2012; Wadsworth and Southwell 2011), the expression of discontent with military modernization and organizational reforms (Dobbs and Do 2019; Heinecken 2014), and veterans calling out harassment and bullying cultures in the armed forces (Wadham 2016) as well as notable whistleblowing of war crimes (Crompvoets 2021). In the Australian case, critiques of Defense led by contemporary veterans and their families argue that the military welfare model is failing, with it being a primary source of unnecessary suffering, high unemployment rates and suicide by those that have served in recent decades (West 2022).

To summarize, by following a traditional conceptualization of cultural militarization and emphasizing the hegemonic power of military institutions, scholars in the sociology of sport has tended to analyze the sport/war nexus in ways that have paid insufficient attention to how it may reflect broader social changes in society. This is despite other sociological fields identifying a growing civilian–military gap in society. To develop a more multidimensional understanding the sport/war nexus, a case study will be undertaken of the 2018 Sydney Invictus Games. As outlined below, this event will be analyzed by drawing on the strong program of cultural sociology, a perspective that has as its strong suit a consistent appreciation of the relative autonomy of culture in reflecting as well as directing social change.

## Methodology

To address the above limitations in the sociological comprehension of the sport/war nexus, I draw on the ‘strong program’ of cultural sociology (Alexander and Smith 2018). This approach attempts to account for the relative autonomy of culture, recognizing how cultural variables have the potential to interfere with and have the power to direct social trends. While the strong program has emerged from neo-Durkheimian cultural theory, it seeks a balanced appreciation of symbolic power in a way that recognizes the need to identify causality in proximate actors and agencies. In addition to contributing to the sociology of sport, the use of the strong program to analyze the Invictus Games is also instructive to the sociology of the military. Analysis in this area is currently dominated by reductionist approaches emphasizing ideological domination and elite interests with far less utilization in this field of otherwise popular methodologies that are culturally sensitive. This includes a reluctance to comprehend the social world from the point of view of ordinary social actors and account for the performative nature of social life (Smith 2005; West and Crosbie 2021).

The thematic analysis of media surrounding the 2018 Sydney Games is drawn from a dataset of the ABC’s coverage during the event. This includes analysis of over 20 hours of live televising of the competition, the opening and closing ceremonies, and all ABC Television content specifically produced on the Games. The latter constitutes some of the ABC’s iconic programs across various genres, ranging from the current affairs program Australian Story, the science documentary series Catalyst, and the popular You Can’t Ask That, a TV series aimed at breaking down stereotypes by members of minority groups answering questions sent in by the public. The dataset also includes 50 online articles of the Games on the ABC news site that were produced during the event. Initially this dataset was viewed and read through with major themes inductively highlighted. Categories were developed for identifying the major themes of the reporting. These were then subject to further coding in a subsequent analysis of the content with specific representations and quotes highlighted for illustration of major themes.

While the dataset is drawn from media broadcast and published during the dates of competition, this at times included coverage of happenings that occurred prior to the competition. This includes the promotion and framing of the event for the media by organizers and sponsors. For example, ABC news reports and current affairs programs shown during competition included reports and footage of politicians such as the Australian Minister for Defence, Marise Payne, who in announcing the seventy-two members of Australia’s 2018 Invictus Team, stated that ‘Training for events like the Invictus Games gives the athletes the opportunity to overcome their physical and mental hardships and focus on what they can achieve post-injury,’ while also proclaiming that this reflected ‘their inherent fighting spirit’ (Department of Defence 2018). Documentaries shown on the ABC during the Games would also display the Australian War Memorial (AWM) marking 100 days until the Opening Ceremony by prominently flying two large banners picturing participant and former commando Garry Robinson. One banner was of Robinson in combat uniform prior to his injuries, suffered from a Blackhawk helicopter crash in Afghanistan during 2010, the other of him dressed in his Australian Invictus uniform (Australian Story 2018). Generally, such promotional media privileged those participants whose injuries relate to deployment, what Caddick et al (2021) terms the ‘hierarchy of wounding.’ As will be outlined below, this often differed from the ‘live’ coverage and commentary of the competition as it performatively played out.

Consistent with the strong program tradition, the cultural analysis undertaken of the data is not one that seeks to discern specific ‘media effects.’ Rather the media analysis in the study is concerned with the use and reimagining of societal level discourses and narratives and the associated meaning-making process as it occurs across different groups and institutions. This is aligned with the ‘deep mediatization’ approach in media analysis (Couldry and Hepp 2017) that emphasizes the blurred boundaries between the mass media and other fields in society. From this perspective the meanings of televised major sporting events derive from a degree of narrative consensus in society and from across multiple media players and platforms, including the digital sphere. The Invictus Games as well as other major sporting occasions are culturally significant in that despite increasing cross-media diversity and fragmentation of audiences, they constitute a contemporary ‘media event’ (Dayan 2008) that culturally interconnect people through acts of communication. In reporting of the Games, specific attention was paid to mention of on-site spectatorship, reflecting the importance of this phenomenon amongst studies in this mediatization and media events tradition (Givoni 2014). Before outlining the results of the thematic analysis, I first outline the more general organizational dimensions and official discourses that surround the Invictus Games.

## The Invictus generation

The Invictus Games is provided cultural legitimacy by the established sport/war nexus, but it also has distinctive origins and characteristics. The Invictus Foundation officially states that Prince Harry was inspired to inaugurate the Invictus Games after having observed the Warrior Games during a trip to the United States in 2013. The Warrior Games had been run annually since 2010 by the US Department of Defense, and is a multi-sport competition for wounded, injured or ill military personnel and veterans. Parasport as a contemporary cultural phenomenon played a significant role in the establishment of the Warrior Games, with the event between 2010 and 2014 held at the U.S. Olympic and Paralympic Training Center in Colorado Springs. The official website of the Invictus Foundation notes that Prince Harry here saw ‘first-hand how the power of sport can help physically, psychologically, and socially those suffering from injuries and illness. He was inspired by his visit and the Invictus Games was born.’

As a multi-nation event and one run by a non-government organization independent of any nation state and separate from any intergovernmental organization, the Invictus Games is perhaps more reflective of the Olympics than the Warrior Games, albeit lacking truly global participation and the type of cosmopolitanism that underpinned Coubertin’s vision. The Invictus Games is also like the Olympics as it is characterized by a sporting invention of tradition, involving the presentation of a social phenomenon as being traditional, and as such normalized through it being connected with a distant past, where in fact it is a relatively recent phenomenon reflecting contemporary cultural mores (Roche 2000). For the Invictus Games this is not through making a connection to historical sporting contests as is the case for the Olympics. Rather its claim to primordialism centers on the word and idea Invictus. Meaning ‘unconquered,’ Invictus is drawn from a poem of the same name written by the Victorian Era poet William Ernest Henley (1888). Invictus was written after Henley had his leg amputated due to complications arising from tuberculosis with the text evoking Victorian era values of stoicism, self-discipline, and fortitude. The motto of the Invictus Games is ‘I AM,’ a phrase appearing repeatedly in the poem: ‘I am the master of my fate, I am the captain of my soul.’ Neo-conservative commentators see these cultural traits as being regrettably weakened with the rise of the welfare state and postmodern sensibilities (Carroll 2004; Furedi 2007). However, Invictus also has a contemporary association with progressive politics through Nelson Mandela popularly believed to have frequently recited the poem while incarcerated at Robben Island during the Apartheid era in South Africa, and in doing so empowering himself and other inmates (Boehmer 2008). For this reason, the 2009 biographical sports drama film on the victory by South Africa in the 1995 Rugby World Cup while Mandela was President, directed by Clint Eastwood and starring Hollywood actors Morgan Freeman and Matt Damon, is titled Invictus. While not directly referenced in the reporting around the Invictus Games, ‘I AM’ (ehyeh in Hebrew) also has a biblical foundation as a statement of independence and faith (e.g., Exodus, 3:14) as well as a Godly proclamation demanding obedience (e.g., Revelation, 1:8). The significance of ‘I AM’ motto for the 2018 Invictus Games is evident with a two meters tall, three-dimensional steel monument of these words being placed at the Games Village for the duration of the 2018 competition. Having been signed by competitors, it was subsequently acquired by the Australian War Memorial as it was believed to have ‘provided daily inspiration to competitors and their families’ (Gist 2018).

Despite the Victorian origins of Henley’s poem and there being well established historical symbolic ties between war and sport, the Invictus Games has a direct concern with contemporary veterans, a group defined by serving during the 20-year long war in Afghanistan and Iraq, conflicts that are characterized by new forms of urban warfare (King 2021) and the rise of suicide bombing (Hassan 2011). It is these veterans that are frequently referred to as the Invictus Generation (e.g., Hayne and Healy 2018). Compared with the World Wars, the Korean War and the subsequent Vietnam/Second Indochina War, the recent Middle East operations are historically distinctive in relation to the relatively low loss of life amongst Western military personnel (e.g., Blum and DeBruyne 2020). However, these conflicts have been subject to far greater levels of public concern and academic and policy recognition around the psychological trauma related to deployment (McFarlane 2015; Winter 2015). Central to this development has been the above-mentioned increased political activism by veterans and their families (Messner 2021).

This rise of individual and societal cultural trauma discourses is significant for comprehending the meanings of the Invictus Games and the support it receives from Defense forces, governments, corporate sponsors, and ex-service organizations. From the perspective of the strong program of cultural sociology, the reporting of individual experiences of psychological trauma and stress can be understood to be symbolically connected to broader societal discourses around war and other mass traumatic events, and to the disruptive nature of social change generally (Alexander 2016). Rather than being a hegemonic field that resists these therapeutic discourses, the below analysis outlines how the Invictus Games draws upon them in establishing its own discourses and narratives. This is facilitated by the Invictus Games having a ritual form that is distinctive from other sporting contests. For example, instead of being underpinned by an ethos of international competitive elitism, the Invictus Games is designed to have a strong utilitarian character with participation being portrayed as therapeutic for recovery from physical and associated psychological injury. This is evident in there being no medal counts and the claimed basis of selection being the role participation plays in recovery. As the official website of the Invictus Foundation (2022) states, the Games attempt to ‘harness the power of sport’ ‘to inspire recovery’ and ‘support rehabilitation.’ However, these are connected to a broader goal, to ‘generate a wider understanding and respect for those who serve their country’ (Invictus Foundation 2022).

This attempt by the Invictus Foundation to address the civil-military gap involves an unprecedented portrayal of the Western warrior’s frailty, including the competition providing a stage to recognize and celebrate a diversity of ordinary serving members of the armed forces. For example, the Invictus Games draws participants not only from the survivors and wounded from war zones but also the everyday service men and women who have been injured or attained illness during a time while they have served, whether this occurred or not as part of their military role. In this way the Invictus Games can be understood as furthering a cultural trend evident from the late twentieth century in war commemoration that seeks to be more inclusive (King 2010). This has seen memorials account for a diversity of military personal beyond those in combat and recognizing not only those that died in war but those that survived (Schwartz and Bayman 1999).

While to a degree the organizational characteristics of the Invictus Games work as a kind of cultural script for the narration of the 2018 Invictus Games, the event also has a strong performative dimension, with meaning-making being created through its enactment by participants, sponsors, the media, and audiences, all having relatively different stakes and interests. Participants are particularly significant for meaning-making in the Games, with the lack of focus on competitive sports nationalism giving a discursive prominence to individual participants, their injuries and rehabilitation stories. In this regard, veterans are likely selected not only in relation to the benefit the Games might provide to their cases of rehabilitation but in how they are more likely than others to represent and embody the Invictus ideal. The meaning of the Games though must also account for the significant support it receives from established custodians of military identity, and actors that have been central to promoting the traditional sport/war nexus, such as national militaries, nation-state Veteran Affairs departments and established ex-service organizations. What discourses and narratives emerge in such an organizational and cultural context, and how might these play out in the 2018 Australian Games?

Accounting for the influence of sponsoring institutions is particularly significant for comprehending the 2018 Games in Sydney as it coincided with the final year of the WWI Centennial. The case is also potentially distinctive with Australian national identity thought to be strongly characterized by traditional symbolic connections between sport and war (Rowe 2017). The militarization of culture in Australia is also widely considered to have increased in recent decades as part of a more entrepreneurial role of the Department of Veteran’s Affairs in commemorating the Anzac (Australia and New Zealand Army Corps) military legend (Lake et al. 2010; Stephens and Broinowski 2017). This includes the rise of popularity and cultural standing of the Australian Football League’s (AFL) annual Anzac Day football match (Fowler 2020; Pascoe 2007). Amongst the major local corporate ‘supporters’ of the 2018 Games were also an array of global arms manufacturers, including Boeing, Lockheed Martin, Raytheon, and SAAB (Invictus Games 2018), whose operations in Australia reflect an unprecedented funding of Defense ‘sovereign capability’ and a move to a One Defence capability model in which the Defense industry is now recognized as a key partner of the military (Department of Defence 2015).

### 2018 Invictus iteration

The article will now outline the dominant discourses and narratives that appeared in the ABC reporting of the 2008 Invictus Games in Sydney. Prince Harry was a central feature in the various television productions for the Games and in news media reporting, with attention particularly paid to his involvement in the origins of the Games as well as in profiling participant experiences of their celebrity meeting of him and his newlywed celebrity actress wife Meghan Markle. While Prince Harry served in Afghanistan, something that is believed to be important in turning around his reputation as a wayward Royal (Jewell 2008), it is his role in initiating the Invictus Games to which he was celebrated. For example, a headline from the Invictus episode of the Australian Story (2018) current affairs series states ‘How Prince Harry “saved” commando Garry’ (Feller 2018). Yet media discourse, even when centered on Prince Harry and Meghan Markle, was also orientated to participants, typically involving the exalting of the competitors. At the Opening Ceremony, for example, the ABC televised news profiled how Prince Harry told the competitors that ‘You are the optimistic generation. You are the new generation of service, and you are the role models to us all’ and that ‘The Invictus generation has chosen to serve their countries in conflicts that are complex and dangerous and far too often this dedication goes unrecognized.’ In the Closing Ceremony Prince Harry would expand on the way participants should be considered role models:

‘Your example goes beyond the military community. It is about more than just your inspiring stories of recovery from injury and illness. It is about your example of determination, of optimism, of strength, honor, and friendship, or as the Aussies call it ‘mateship,’ as a core value that has the power to inspire the world. That is something we can all aspire to…. the Invictus example. You can be a teacher or a doctor, a mum or a dad, a child or a grandparent, a farmer, a plumber, a lawyer, or a CEO. Or anything at all. You can identify something in your own life that you want to change for the better. And you can let the men and women of the Invictus Games remind you that no challenge is too difficult to overcome.’

This status reversal of the disabled and the abled marks the Games as a liminal rite in Victor Turner’s (1974) terms, albeit one without a conventional carnivalesque character. The focus on relative ordinary military personnel and a forgotten group in society also has a status reveal effect, distinguishing the Invictus Games from traditional military remembrances where the emphasis is on foundation moments, historical continuity and particular individuals who have shown extraordinary bravery and courage. In contrast, media reporting of the Games included prominent competitor profiles of those suffering from injuries and illness unrelated to service. For example, the ABC documentary on wheelchair rugby (Cone 2018) and the popular You Can’t Ask That (2018) segments prominently profiled participants suffering from Stage Two glioma, multiple sclerosis and a car accident.

The unusual prominence of ordinary military personnel and veterans in the Games is at times acknowledged by the competitors themselves. For example, Matt Brumby, who co-captained the Australian team and who sustained a spinal injury during a clearance diver selection course, recalls in the ABC documentary on the wheelchair rugby team that ‘I had a conscious thought of ‘should I be here’? …you know, these boys are special forces’ (Cone 2018), referring to participants Garry Robinson and Pete Rudland. When such ex-members of the Special Forces are profiled in media portrayals, it typically involves them having a history of sporting activity and success prior to their injury, something which can be recreated through the Invictus Games. Discourses of competitive elite sports is also evident in a variety of stories around participants having gone on from their involvement in the Invictus Games to represent Australia at the Paralympic Games and in other elite adaptive sports competitions (e.g., Giles 2018). However, in most cases, as outlined in the quotes below, it is the lack of a prior sporting pedigree by participants that received media attention with this being an important part of the Invictus Games narrative as it relates to the healing power of sport.

‘I remember thinking to myself, I now have this new door opening which I could explore. It was a new opportunity that gave me something to aim for…one I found out that “you have a spot in the trials in January” last year it was “oh no”, I better start training for these things… once I learnt I was on the team, it’s like, wait, “I’m an athlete?, “I’m going to be representing Australia?”’ (Nathan Parker). (You Can’t Ask That 2018)

‘You ask all my friends that I have ever had, most of them can’t believe I can do sport. So I was rather intoxicated at the time when I filled out the application. A couple of weeks later I get an email saying I’ve been selected to try out for the team… of course it was I had to put down that can of Coke and step away from that Scotch bottle and find the local gym and sign up… it’s kind of like a joke. There I was in the Green and Gold, how funny. Cop that one PE teacher’ (Stewart Sherman). (You Can’t Ask That 2018)

This post-heroic character of the Invictus Games is also evident in participants encouraging audiences to look beyond the façade of the traditional heroic portrayal of the warrior, emphasizing that appearances often differ from what goes on beneath the surface.

‘A lot of people probably look at me and go "what’s wrong with you? You look fine"! But that’s the challenge with mental health stuff… you can (only) keep that mask on for so long…’ (Trudy Lines). (Cone 2018)

‘Because you look so normal, people struggle to think well why can’t you work? What’s wrong with you, you know? What do you mean, what do you mean you are feeling depressed or whatever’ (Danny Jeffrey). (You Can’t Ask That 2018).

Whereas narratives about overcoming adversity are prominent in relation to traditional sports nationalism, these typically involve accounts whereby individuals have already overcome challenges, having raised themselves up to a high level of achievement. In contrast, the Invictus Games is itself portrayed as the opportunity and mechanism by which hurdles can be overcome. However, as illustrated in the below quotes, it is the attempt and effort at recovery rather than enduring success that is typically championed. For this reason, participants are often openly portrayed as experiencing ongoing difficulties. This is most common for profiles of those participating in their first Invictus Games, but it is also the case for various participants that are chosen for multiple Games, despite this practice being difficult to comprehend with the above-mentioned selection policy.

‘Most days I wake up feeling like less of a man… I feel very inadequate against, you know, my friends, my family, that I shouldn’t be around them or shouldn’t get out of bed in the morning because it is just not worth it’ (Brandon Griffiths). (Cone 2018)

‘At the moment my life, it’s a wreak. Training for the Invictus Games, it has given me a purpose. It’s given me motivation, something to strive for and something to train for. If I want to be selected, I have to leave the house and train. So it gets me out of bed in the morning. For me to make the team it would be amazing’ (Matthew Blunt). (Cone 2018)

The perceived healing power of the Invictus Games and how it differs from more mainstream international sporting competitions, including the Paralympics, is also illustrated in the awards given at the Closing Ceremony of the Games. For example, the winner of the Above and Beyond award in 2018 was the Netherlands’ Edwin Vermetten who during competition in the wheelchair tennis mixed doubles tournament provided emotional support to his partner when they had a reoccurrence of Post-Traumatic Stress during the match, triggered by a helicopter flying overhead.

This qualitative dimension of the Invictus Spirit that focuses on the manner and disposition to living with injury, disability, and illness, however, is also what facilitates the application of neoliberal ideological frames. This is done not only by displacing state responsibility for health and welfare onto the private individuals but by discouraging political criticism through the advancement of a culture which encourages reflexive biographical constructions that insist on emotional positivity. As expressed by Prince Harry at the 2018 Closing Ceremony, in ‘a world where negativity is given too much of a platform,’ the competitors ‘want to live, rather than just be alive.’ He goes on to state that ‘Our competitors have helped turn the issue of mental health from a sad story to an inspiring one.’ This Invictus spirit draws on sporting discourses of individualistic empowerment and emancipation, as has been romanticized in relation to minority groups by corporations such as Nike (Helstein 2003; Hoffmann et al. 2020). This hyper-individualism expressed through the Invictus Games places no responsibility or allows any attention to be drawn to inadequacies with military organization or military welfare policies and practices (Rembis 2013, p. 128).

When factors external to the individual are recognized in relation to recovery and rehabilitation it is not in relation to national institutions, such as veteran health and welfare systems, but support provided by fellow veterans, families, and audiences at the Games. The connection between these three sources of support and patriotism is indicated by Australian journalist Chris Bath who describes that from what she has seen, the ‘Invictus Games celebrate love. Love of your country, love of your fellow man, the love of family and friends’ (Bath 2018). In this narrative each participant is seen as not only engaged with addressing their own demons but providing support and being an inspiration to others, with their participation in the Invictus Games being portrayed as a selfless act. As ex-Commando Garry Robinson states, ‘If I can save one person, I don’t care who it is, where it is, what it is, I want to inspire one person and I know my journey, for me will be complete’ (You Can’t Ask That 2018).

However, it is not simply the participants who are the focus of media reporting but also their family members, with considerable attention given to the support provided by partners and parents. In this discourse family support is frequently portrayed as the crucial factor behind extraordinary recovery, often framed in contrast to official medical diagnosis and expert advice. This is clearest in the Closing Ceremony when Meghan Markle spoke about the Novak family from Chicago:

‘When their son Ryan suffered a severe injury leaving him paralyzed from the waist down, doctors said he would never be able to walk again. But after speaking to his mum Kerry, it was clear it was through Ryan’s strength of spirit and through the unwavering support of his parents he was able to prove all those doctor’s wrong…’

The children of competitors, as indicated in the news article quote below, are frequently narrated in this regard, with the performance of a parent re-establishing traditional lines of family respect and role modeling.

‘Danyan Jones, 13, has travelled from Ballina in northern NSW to watch his stepfather compete in wheelchair rugby and tennis. "It's really proud watching him because when I watch him I feel like that's what I want to do," Danyan said. "When we first met him, I would always say 'I want to join the army when I grow up'." For Danyan, the transformation Invictus has wrought in his stepfather is clear, and a huge relief. "It's a really good feeling because before he started Invictus he would just hide in the wardrobe," he said. "But then he started going to Invictus and he's out playing wheelchair rugby and wheelchair tennis some weekends and yeah — it's a really big relief to watch him do that".’ (Tatham 2018)

The significance of the audience to the Invictus narrative is not limited to peers and family. Rather the perceived healing power of sport and wellbeing of participants is also portrayed as being heavily dependent upon the public attending the competition and viewing the televised broadcast. This is illustrated in the following quote from the Sydney Invictus Games CEO, and recently transitioned veteran, Patrick Kidd. He describes how audiences should appropriately view the Games. Rather than gazing upon participants for the purposes of entertainment, he stresses it is a more serious endeavor where ‘by watching, by being interested in them, by understanding what they're about is that you, yourself, can understand better what it means to serve in the nation's military and how we can all support them as we go forward’ (NSW Government 2018).

How do such accounts differ from other glorifications of sport as character building and providing an integrative effect for society? Sport in the West is popularly used to address a range of social problems and the individual empowerment narratives prominent in reporting of the Invictus Games are also found in parasport broadly. However, the Invictus spirit is distinctive in that its story of rehabilitation is not in sport facilitating the development of a new social identity and social networks, but rather a mechanism for participants to recapture and reengage with their former military warrior selves and exalt public respect for the armed forces. These accounts typically involve veteran suffering leading them away from the institutionalized military identity they previously held prior to injury or illness, with the Invictus Games facilitated rehabilitation coming via them rediscovering their former warrior selves. In this way the Invictus Games is portrayed not only involving a physical rehabilitation for the ills that individual veterans suffer but more broadly having a role in a cultural rehabilitation for participants. This transformation is then held up as a model for society at large, one in which contemporary culture can rehabilitate itself from the circumstances that have resulted in the current civil–military gap.

This meta narrative though is metaphorically played out at the micro level through participants’ comprehensions of the relevance of the Invictus Games and sport for their recovery. One prominent way this occurs is by sport being portrayed as a surrogate for the former military lives of participants, whether that be in providing an alternative way for participants to represent their nation in uniform or attain the sort of comradeship they enjoyed in their service. As representative of this coverage, consider these quotes from You Can’t Ask That (2018).

‘When the opportunity arose to represent my country again the old Garry came back. All those old traits. I obviously didn’t look the same or compete the same but the old Garry was there and he helped me get to where I am today’ (Gary Robinson).

‘Invictus makes them feel like their family again. That they are home. Because once your discharged you lose that family’ (Sonya Newman).

‘I’ve heard so many guys and girls say this is just like being back in. It will be that sense of mateship, that sense of pride, belonging, belonging is a big big one that you suffer from, a perceived lack of belonging’ (Stewart Sherman).

‘The whole time in our military careers we all wore the Australian flag on our left sleeve. It’s that chance again to, ok, yep, still achieve things and still wear that flag and represent my country, just in another way’ (Danny Jeffrey).

This reporting often coincided with participants expressing a nostalgia for their prior military service, including that they have no regrets in joining the military, despite nearly all being medically discharged or in the process of exiting the military as they are not deemed fit for service.

## Contemporizing service and disarming contemporary veterans

The Invictus Games can be thought of as a kind of status elevation ceremony (Rouse 1996; Garfinkel 1956) for current serving military personnel and veterans, one that attempts to hold participants up as role models for the population and in doing so exalt the armed forces generally. This is in part the attraction of the Invictus Games for the traditional custodians of military identity. However, conventional conceptualizations of the sport/war nexus did not dominate the discourse of the 2018 Sydney Games. The discursive construction of the Invictus spirit in many ways also countered the organizational culture of the military, one that is characterized by doctrine and tradition. Recognizing participation and the conditions of ordinary members of the armed forces independent of achievement is certainly in tension with the hierarchical organization of professional militaries and their system of honors and awards (Carter 2021). This is not surprising as the Invictus spirit in essence is about using the cultural codes of identity politics to champion military institutions and worldviews in ways that gives it a contemporary cultural relevance in a world where these are otherwise being diminished through a growing civil–military gap and post-heroic conceptualizations of military service.

In the context of identity politics, the military veterans in the Invictus Games are framed as a distinctive and marginal group in society rather than being representatives of the national ideal in a traditional modern way. Rather than being the contemporary embodiment of military tradition, this identity politics framing imagines the military as a kind of neo-tribe. This is consistent with the rhetoric around an Invictus generation and the post-heroic willingness to discursively focus on ordinary Invictus participants suffering from more everyday ailments and stresses. Such representations feed into a cultural politics of recognition, one in which traditional as well as new Defence associations and groups can demand public respect for contemporary veterans and service, as to withhold it would be to inflict real harm on this group cast as a minority and victim (cf. Twomey 2013). In the context of the Invictus Games this harm is not merely psychological and emotional, but physical as recognition for participants in the Games is portrayed as critical for rehabilitation. In this way, despite the media portrayal of the Games breaking with heroic cultural traditions related to the sport/war nexus, these emergent discourses and narratives are generally accepted by traditional stakeholders of military identity as they are seen as being used to redress the civil–military gap in society.

The attraction of the Invictus Games for the custodians of military identity is also that it promotes healthism (Aamann 2020), with the problems and solutions related to veteran health comprehended at the level of the individual, their personal networks and the recognition provided by the public. Healthism in the Games draws on contemporary neoliberal discourses around emotional positivity as the basis of empowerment. This discursive enactment was facilitated by healthism being prominent in sporting discourses and specifically, as it relates to disability, in the Paralympics. Healthism was also naturalized by a romantic primordial construction of Invictus related to Victorian Era’s notions of individual will, stoicism, and fortitude. Nostalgia though also plays a broader role in social constructing the Invictus spirit through rehabilitation being framed in the context of a loss of a bygone era in which not only is military service highly respected and acknowledged by society, but the challenges of life could be managed without the support provided by bureaucratic health and welfare systems. In this longed-for age, care comes from traditional family structures and tight peer relations, something the Invictus Games promotes as being strengthened through a viewing or shared experience of physical and psychological challenge. However, what is crucial for such representations as it relates to militarization processes is that cases of successful physical and psychological recovery are explained by participants reembracing their warrior identities that were held when serving in the armed forces. This assignment of agency allows for military life to be both culturally contemporized in the context of identity politics but also championed in ways that appear relevant for the identity challenges of today. This is despite the otherwise vast amount of media attention and academic studies on the difficulties that veterans face, both during service and in military–civilian transition.

This romantic framing of military life explains the silence in the data around the political activism by veterans and their complaints regarding the systemic failures of the veteran health and welfare system as well as the social problems that derive from the nature of military organization. While the discourses and narratives of the Invictus Games are framed as a response to a civil–military gap in Western societies, and specifically the lack of civilian societal recognition of military service, nothing is mentioned in the dataset about the failure of cultural recognition for veterans coming from within Defense itself and how this can be adverse for veteran wellbeing. Mainstream media has otherwise been supportive and widely covered contemporary veteran activism on topics such as the lack of support given by militaries to veterans in their transition to civilian life, with a particular concern for high veteran unemployment and suicide rates amongst this group being the consequences. In the context of the Invictus Games’ utilization of identity politics such critical media discourses largely disappear.

The existing scholarly perspective on the sport/war nexus would certainly point to the ways in which the reporting of the Invictus Games champions military veterans and fails to acknowledge systemic problems with military organization and culture. However, scholars working in this tradition typically see sport as a vessel that is filled at will with traditional military narratives. As such it is unlikely that such a study would give much attention to the prominent role of identity politics rhetoric in the narration of the Invictus Games and the prominent and innovative strategic use of post-heroic narratives. Unlike the dominant militarization thesis that see military needs as being reflected in dominant cultural representation and public consciousness, this article has argued that the discourses and narratives of the Invictus Games should be comprehended as a reaction to social change in the West and an associated de-militarizing of the civilian sphere. Utilizing the strong program of cultural sociology, a paradigm that seeks to appreciate the relative autonomy of culture, this article was able to identify the ways social actors in Defense have both been influenced by as well as attempted to utilize new civilian cultural codes. In the media coverage of the Invictus Games there is a hegemonic championing of military life, but unlike most studies of mainstream sporting contests analyzed in the sociology of sport, this has not come about through traditional heroic narratives of the sport-war nexus but post-heroic discourses in which the frailties of military personnel are recognized. This is done to emphasize romantic sporting notions of individual empowerment and as part of a call for a return to traditional  civil–military order.

Such discourses resonate because they are more sensitive and consistent with the contemporary civil–military gap, yet they champion the military in ways that can advance militarization. This militarizing effect of the Invictus Games requires a more multidimensional comprehension of the interconnections between the military and civilian spheres than is typically appreciated in studies of the sport/war nexus. As outlined above, recent critics have rightly pointed out that the dominant conceptualization of militarization lacks adequate acknowledgment of the way social actors can creatively use and interpret military symbols and narratives, including which resist, reframe, and over-code their intended meanings (West and Crosbie 2021; McKay 2013; Woodwood et al. 2017). While at the discursive level the Invictus Games may advance militarization, it can be thought of as an exceptional case to the dominant sport/war nexus. However, there is also the possibility that the Invictus Games illustrates an emergent social construction of the military that has significant transformative consequences. As outlined above, it is not merely that the cultural framing of the military in the Invictus Games marginalizes critique of the armed forces but by militarizing identity politics and applying the culture of recognition to veterans the Invictus Games encourages empathy for current serving military personnel and portrays military life in ways that gives it a new generational cultural relevance. While it is likely that the transformative potential of such discursive shifts is limited by the current levels of political activism amongst veterans, these counter-narratives could quite rapidly be reduced by organizational reform in the armed forces and veteran welfare systems. In that case there would be fewer barriers to the affective post-heroic yet romantic construction of the contemporary veteran and their service that is evident in the Invictus Games.

## Conclusion

Amongst rising global security tensions and in the wake of prolonged conflicts in Afghanistan and Iraq, it is important for sociology to critically consider the adequacy of existing analytic understandings of the contemporary relationship between war, the military, and civilian culture. Sport is historically a significant arena in which the military has been romanticized and the militarization of society enacted. For this reason, scholars are rightly concerned about sport as a basis for the military to have a profound influence on everyday social practices and in the shaping of collective identities. However, the militarization of culture thesis that has fundamentally informed sociological understanding of the sport/war nexus, has tended to reify the military in ways that has hampered appreciation of the shifting and multidimensional relationship between war, the military, and the civil sphere.

In analyzing the media depiction of the Invictus Games, a recently founded but already culturally significant international sporting event involving military veterans who have become either wounded, injured or sick during their period of service, this article has argued that we need to be critically aware of the way new discourses and narratives are constituting the sport/war nexus. Rather than the Invictus Games being dominated by traditional heroic discourses and narratives that sociologists of sport have long analyzed, the study highlights how the media portrayal of the 2018 Invictus Games involved new discourses and narratives that drew on the rhetoric of identity politics. In this arena a completely new cultural pattern was not required, with parasport and the Paralympics providing a cultural template for connecting the politics of recognition with disability and sport. In the Invictus Games this informed key dimensions of its organization, including a selection policy promoted as inclusive and a focus on participation for rehabilitation rather than competition and results. However, in the performative narration of the Invictus Games, identity politics was heavily drawn upon in ways that sought to champion the military and enact militarization. This allowed for the Invictus Games to be culturally framed in ways that are consistent with the contemporary civil–military gap in Western societies while also seeking to redress it.

Specifically, identity politics in the Invictus Games worked to exalt the armed forces by military veterans being portrayed as a kind of neo-tribe or minority, with traditional patriotic sentiment and cultures of respect for the military being infused with discourses that surround the politics of cultural recognition, those that are typically associated with respect for cultural diversity and human rights. A key dimension of the way that the Invictus Games discursively attempts to rescue the cultural relevance of military life is through a post-heroic celebration of the military’s warrior ethos. Rather than being championed in relation to heroic performances in combat, the Invictus Games exalts warrior identity by portraying it as the resource that participants draw on in attempting to overcome the physical and psychological challenges of rehabilitation and transition, and the challenges of contemporary society generally. This ‘heroism’ is dominant in the coverage of the Games, and within a cultural frame of nostalgia is the basis for organizers and commentators to hold up participants as role models for society at large.

As the study has only been concerned with discursive representation it cannot say how such media portrays may be influential in shifting current attitudes and beliefs in Western societies to military service. However, it is clear from the media analysis that the Games did marginalize otherwise prominent contemporary critiques of the military life and veteran services, activism which is often led by contemporary military veterans. It is likely that the strong institutional support the Invictus Games has received from governments, militaries, and corporate sponsors, is in part related to this silencing effect. In the Australian case, these critiques of Defence institutions center on the difficulties faced by military personnel in successfully transitioning to civilian society following their service. As the dominant narrative of the Invictus Games surrounds the benefits veterans can attain by reengaging with their service identity, there is a danger that the effect of the Invictus Games is that it works against the consistent recommendations of the numerous reports and inquiries into transition, that military personnel need to be better prepared and supported in transiting to civilian life.
